# Using machine learning to improve risk prediction in durable left ventricular assist devices

**DOI:** 10.1371/journal.pone.0247866

**Published:** 2021-03-10

**Authors:** Arman Kilic, Daniel Dochtermann, Rema Padman, James K. Miller, Artur Dubrawski

**Affiliations:** 1 Division of Cardiac Surgery, University of Pittsburgh Medical Center, Pittsburgh, PA, United States of America; 2 Carnegie Mellon University, Pittsburgh, PA, United States of America; Thomas Jefferson University, UNITED STATES

## Abstract

Risk models have historically displayed only moderate predictive performance in estimating mortality risk in left ventricular assist device therapy. This study evaluated whether machine learning can improve risk prediction for left ventricular assist devices. Primary durable left ventricular assist devices reported in the Interagency Registry for Mechanically Assisted Circulatory Support between March 1, 2006 and December 31, 2016 were included. The study cohort was randomly divided 3:1 into training and testing sets. Logistic regression and machine learning models (extreme gradient boosting) were created in the training set for 90-day and 1-year mortality and their performance was evaluated after bootstrapping with 1000 replications in the testing set. Differences in model performance were also evaluated in cases of concordance versus discordance in predicted risk between logistic regression and extreme gradient boosting as defined by equal size patient tertiles. A total of 16,120 patients were included. Calibration metrics were comparable between logistic regression and extreme gradient boosting. C-index was improved with extreme gradient boosting (90-day: 0.707 [0.683–0.730] versus 0.740 [0.717–0.762] and 1-year: 0.691 [0.673–0.710] versus 0.714 [0.695–0.734]; each p<0.001). Net reclassification index analysis similarly demonstrated an improvement of 48.8% and 36.9% for 90-day and 1-year mortality, respectively, with extreme gradient boosting (each p<0.001). Concordance in predicted risk between logistic regression and extreme gradient boosting resulted in substantially improved c-index for both logistic regression and extreme gradient boosting (90-day logistic regression 0.536 versus 0.752, 1-year logistic regression 0.555 versus 0.726, 90-day extreme gradient boosting 0.623 versus 0.772, 1-year extreme gradient boosting 0.613 versus 0.742, each p<0.001). These results demonstrate that machine learning can improve risk model performance for durable left ventricular assist devices, both independently and as an adjunct to logistic regression.

## Introduction

Recent studies have demonstrated an increasing number of durable left ventricular assist devices (LVADs) being implanted in the United States with improving outcomes [1–3]. Despite these trends, there remains no widely utilized risk stratification tool for LVAD therapy. A prior analysis evaluating the predictive performance of the HeartMate II Risk Score, the Model for End-Stage Liver Disease, and the Destination Therapy Risk Score in estimating 90-day mortality risk after durable LVAD implantation demonstrated area under receiver-operating-characteristic curves (AUROC) or c-indices of only 0.59–0.64 in validation cohorts, for example [4]. Machine learning (ML) has been shown to improve risk prediction in cardiac surgery and its use in LVAD therapy remains largely unexplored [5, 6]. The aim of this study was to evaluate whether ML can improve risk prediction in patients undergoing LVAD implantation.

## Materials and methods

### Data source

The data source for this study was the Interagency Registry for Mechanically Assisted Circulatory Support (INTERMACS). INTERMACS is a North American registry of adults who have been implanted with a mechanical circulatory support device for advanced heart failure that has been approved by the Federal Drug Administration. The registry was obtained via the National Heart, Lung, and Blood Institute Biologic Specimen and Data Repository Information Coordinating Center (NHLBI BioLINCC). INTERMACS continues to collect and analyze data through the Society of Thoracic Surgeons but at the time of the data being locked and provided to BioLINCC, there were 170 active centers in the United States and Canada reporting to the registry. The current study was approved by the institutional review board at the University of Pittsburgh. The data were fully anonymized before accessing them and the requirement for informed consent was waived.

### Study population

Adults aged 19 years or older undergoing primary durable LVAD implantation in the INTERMACS database were included. The study period extended from March 1, 2006 to December 31, 2016, with follow-up data available through December 31, 2017. Patients undergoing concomitant valve procedures, coronary revascularization, or right ventricular assist device (RVAD) insertion were also included. Pediatric patients were excluded from analysis as were patients undergoing pump exchange.

### Primary outcomes and variables

The primary outcomes for which risk models were developed included 90-day and 1-year overall mortality. Data included demographics, comorbidities, laboratory parameters, clinic visit measurements, interval events during hospitalization prior to LVAD insertion, and concomitant operative procedures. Post-LVAD data collected in INTERMACS includes adverse events and survival. Clinic visits are scheduled at 1 week, 1 month, 3 months, 6 months, and every 6 months thereafter following implantation.

### Development of risk models

Only patients with adequate follow-up were included in the analyses. INTERMACS stops collecting data once a patient is transplanted and therefore those patients bridged to transplantation were excluded if they were transplanted before 90-days or 1-year in those respective models. Patients who were alive but did not have follow-up to 90-days or 1-year were similarly excluded due to inadequate follow-up. All causes of mortality were included in the mortality analysis. The study cohort was randomly divided in a 3:1 fashion into training and testing sets. For both modeling approaches, only pre-implant variables were considered. Those variables with >10% missing data were excluded.

### Logistic regression models

For the logistic regression (LR) models, univariable LR analysis was conducted to evaluate the association between each individual variable and the outcome of either 90-day or 1-year mortality in the training set. Continuous variables were modeled in a continuous fashion without categorization. Those variables associated with the outcome in univariable analysis with an exploratory p-value less than 0.05 as well as those variables with biologic plausibility were entered into the multivariable LR model in a forwards and backwards stepwise fashion. The year of implantation was included in the models to account for temporal changes.

### Machine learning models

For the machine learning (ML) models, the algorithm that was utilized was extreme gradient boosting, or XGBoost. This is an ensemble ML algorithm that iteratively builds stronger models using a collection of short decision trees. Similar to the LR models, only preoperative variables were considered for inclusion in the ML models. The year of implantation was included in the ML models as well to account for temporal changes. The algorithm identifies optimal split-points in continuous variables to maximize information gain. Non-binary categorical data were one-hot-encoded ensuring no linear dependencies between columns. Tuning of hyperparameters was performed in the training set as part of the model derivation process. Optimal hyperparameters were selected based on those associated with optimal model performance. Permutations of the following parameters were iteratively evaluated for the ML models: learning rate (0.01, 0.1, 0.5, or 1.0), maximum depth of each tree (1, 2, 3, 5, or 10), number of trees (100, 200, 400, 800, or 1600), alpha (0, 1, 3, 5, or 7) and lambda (0, 1, 3, 5, or 7).

### Model performance

Bootstrapping was performed with 1000 replications. This translated to performing the random 3:1 split for training and testing sets 1000 times to obtain average model metrics. Calibration of the models were compared using standardized measures [7]. This included visual calibration plots, observed-to-expected ratios based on decile of risk, calibration-in-the-large or y-intercept of the calibration plot (optimal value of 0), and slope of calibration curve (optimal value of 1). The discriminatory power of the models was measured using the AUROC, or c-index. Average c-indices across the 1000 bootstrap replications were then compared between the LR and ML models for both 90-day and 1-year mortality. This average was derived from the 1000 individual AUROCs obtained for each of the 1000 testing sets generated in the bootstrapping process. The net reclassification index (NRI) was also assessed to evaluate the percentage improvement in identifying both positive and negative cases with the ML models as compared to LR [8].

### Concordance analysis

For the concordance analysis, patients were divided into equal size tertiles based on predicted risk of 90-day or 1-year mortality using the LR models. This was used to categorize patients as low, intermediate, or high risk for 90-day or 1-year mortality. The predicted risk of these outcomes at the individual patient level was then assessed with the ML models, and concordance between LR and ML models was defined as agreement in predicted risk according to the risk thresholds for the low, intermediate, or high risk groups. In other words, concordance occurred when both LR and ML models categorized risk of 90-day or 1-year mortality as low, intermediate, or high. When LR assigned a different level of risk category as compared to ML, the models were defined as discordant. The above calibration and predictive metrics of the models were compared between concordant and discordant cohorts. All statistical analyses in this study were performed using STATA version 14 software (StataCorp, College Station, Texas) and Python programming software.

## Results

### Baseline characteristics of the study cohort

A total of 16,120 patients underwent durable primary LVAD implantation during the study period and met inclusion criteria for the study. Mean age was 57 years and the majority were males (**Table 1**). Non-ischemic and ischemic cardiomyopathy were the most common etiologies of heart failure and were present in roughly the same proportion. Most patients were INTERMACS profile 2 or 3, with 17.8% being INTERMACS profile 1. Comorbidities included chronic kidney disease (16.4%), atrial arrhythmias (14.8%), severe diabetes mellitus (7.1%), pulmonary disease (6.7%), peripheral arterial disease (3.6%), and prior stroke (2.7%). Internal cardioverter defibrillators were in place in the majority at the time of LVAD implantation (78.4%). Destination therapy versus bridge or likely bridge to transplant represented roughly equal portions of the study cohort (**Table 1**). Most patients were supported on intravenous inotropes in the 48 hours prior to LVAD surgery (81.7%), with 22.7% bridged with an intra-aortic balloon pump. The most frequent concomitant procedures included tricuspid valve repair (13.9%) and atrial septal defect or patent foramen ovale closure (7.3%).

**Table 1:** Baseline characteristics of patients undergoing left ventricular assist device implantation in the study cohort.

	Study Cohort (n = 16,120)
Age (years)	57.0 ± 13.1
Female	3,512 (21.8%)
Caucasian Race	10,786 (66.9%)
Body Mass Index (kg/m^2^)	28.9 ± 7.3
Blood Type	
O	7,855 (49.4%)
A	5,607 (35.3%)
B	1,939 (12.2%)
AB	501 (3.2%)
Severe Diabetes Mellitus	1,136 (7.1%)
Pulmonary Disease	1,078 (6.7%)
Chronic Kidney Disease	2,637 (16.4%)
Peripheral Arterial Disease	572 (3.6%)
Prior Stroke	440 (2.7%)
Current Smoker	614 (3.8%)
Provider-Assessed Frailty	796 (4.9%)
Atrial Arrhythmia	2,384 (14.8%)
Liver Dysfunction	482 (3.0%)
History of Alcohol Abuse	886 (5.5%)
History of Drug Use	844 (5.2%)
History of Malignancy	624 (3.9%)
Pulmonary Hypertension	2,566 (15.9%)
Severe Depression	318 (2.0%)
Prior CABG	3,590 (22.3%)
Prior Valve Surgery	1,283 (8.0%)
Internal Cardioverter Defibrillator	12,635 (78.4%)
Device Strategy	
Bridge to Recovery	122 (0.8%)
BTT (already listed)	3,532 (21.9%)
BTT (listing likely)	2,733 (17.0%)
BTT (listing moderately likely)	1,573 (9.8%)
BTT (listing unlikely)	526 (3.3%)
Destination Therapy	7,543 (46.8%)
Other	91 (0.6%)
Events in the 48 Hours Prior to	
LVAD Implant	
Dialysis	322 (2.0%)
Mechanical Ventilation	1,262 (7.8%)
Intra-Aortic Balloon Pump	3,665 (22.7%)
ECMO	531 (3.3%)
Intravenous Inotropes	13,168 (81.7%)
Etiology of Heart Failure	
Non-ischemic Cardiomyopathy	7,632 (47.8%)
Ischemic Cardiomyopathy	7,460 (46.7%)
Post-Partum Cardiomyopathy	265 (1.7%)
Valvular Heart Disease	197 (1.2%)
Hypertrophic Cardiomyopathy	111 (0.7%)
Restrictive Cardiomyopathy	208 (1.3%)
Congenital Heart Disease	94 (0.6%)
INTERMACS Profile	
1	2,872 (17.8%)
2	5,734 (35.6%)
3	4,940 (30.7%)
4	2,024 (12,6%)
5	334 (2,1%)
6	139 (0.9%)
7	77 (0.5%)
Serum Creatinine (mg/dL)	1.25 ± 0.5
Serum Sodium (mmol/L)	134.6 ± 8.3
INR	1.22 ± 0.64
Serum Albumin (g/dL)	3.08 ± 1.14
NYHA Class IV	12,429 (77.1%)
Concomitant Procedures	
ASD/PFO Closure	1,179 (7.3%)
Aortic Valve Repair	248 (1.5%)
Aortic Valve Replacement	369 (2.3%)
Mitral Valve Repair	516 (3.2%)
Mitral Valve Replacement	66 (0.4%)
Tricuspid Valve Repair	2,246 (13.9%)
Tricuspid Valve Replacement	145 (0.9%)
Coronary Artery Bypass Grafting	271 (1.7%)

*Abbreviations*: ASD, atrial septal defect; BTT, bridge to transplantation; CABG, coronary artery bypass grafting; ECMO, extracorporeal membrane oxygenation; INR, international normalized ratio; INTERMACS, Interagency Registry for Mechanically Assisted Circulatory Support; LVAD, left ventricular assist device; NYHA, New York Heart Association; PFO, patent foramen ovale.

### Model performance

**Fig 1:**
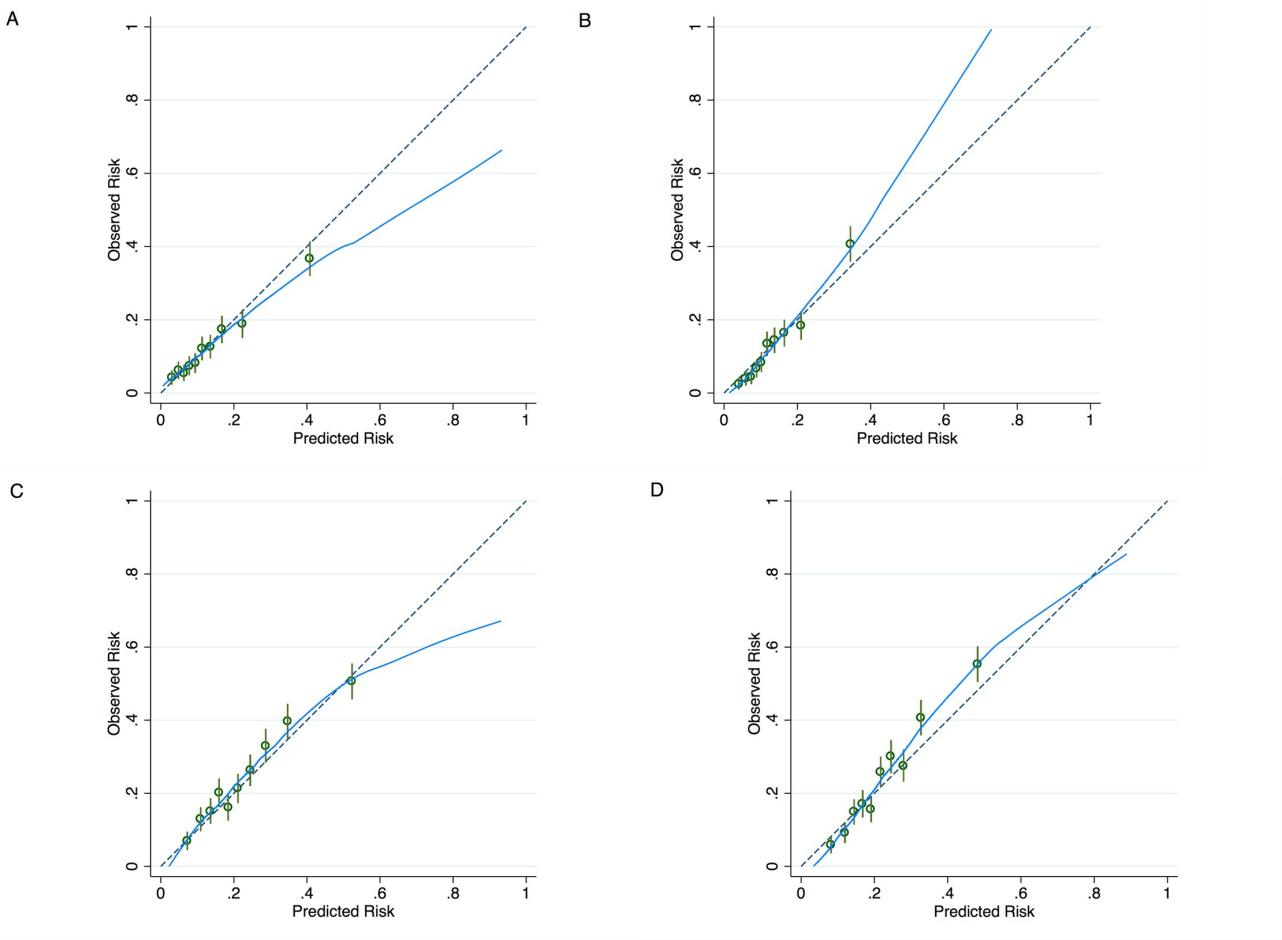
Calibration plots for (A) logistic regression model for 90-day mortality, (B) machine learning model for 90-day mortality, (C) logistic regression model for 1-year mortality, and (D) machine learning model for 1-year mortality.

**Fig 2:**
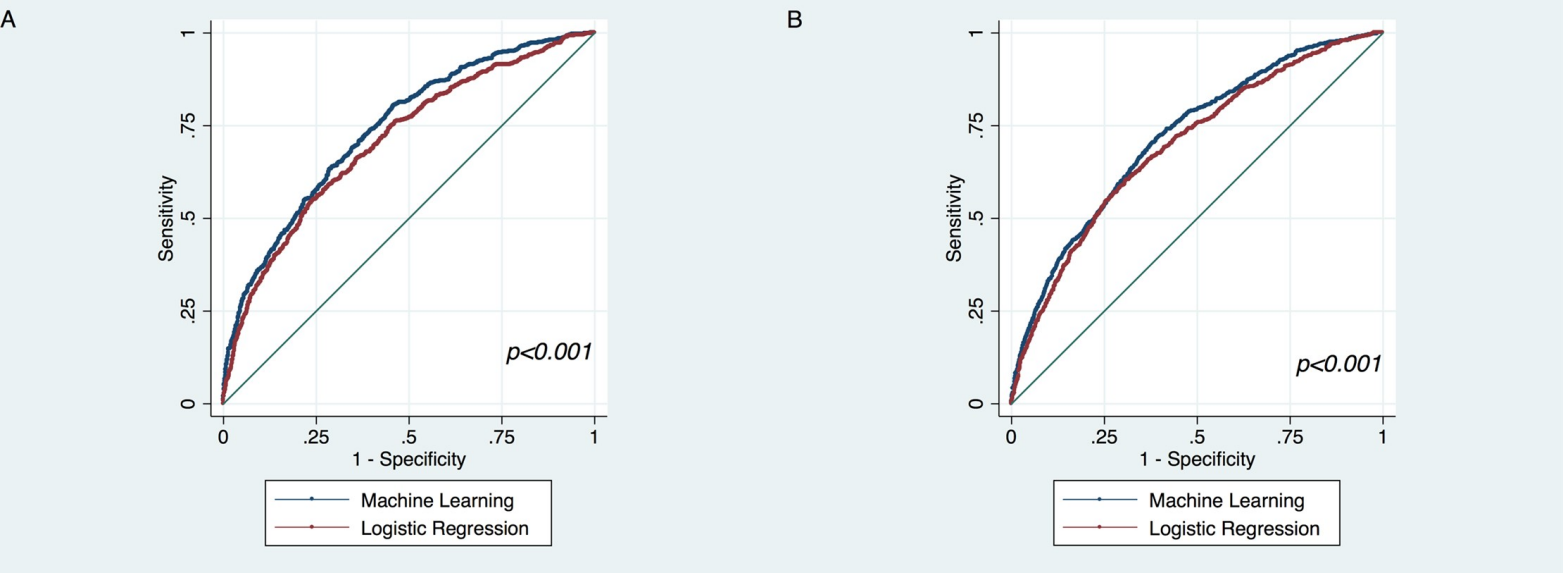
Comparison of area under receiver operating characteristic curves for (A) 90-day mortality and (B) 1-year mortality between the logistic regression and machine learning models.

90-day mortality occurred in 13.3% (n = 2,144) and 1-year mortality in 23.1% (n = 3,718). For both 90-day and 1-year mortality, the LR and ML models were well calibrated with metrics close to optimal values (**Table 2 and Fig 1A–1D**). A comparison of average AUROC demonstrated improvements with ML as compared to LR for 90-day mortality (0.707 versus 0.740, p<0.001) as well as 1-year mortality (0.691 versus 0.714, p<0.001) (**Table 3 and Fig 2A and 2B**). NRI analysis also demonstrated significant improvements in predictive capability with ML. Moreover, there was an improvement of 48.8% (p<0.001) in 90-day mortality prediction and 36.9% (p<0.001) improvement in 1-year mortality prediction with ML in NRI analysis. There was some degree of variation across the bootstrap replications in features that were most important in the ML models. Risk factors that were commonly most important in the 90-day mortality ML model included number of significant interventions during the current hospitalization prior to LVAD surgery, INTERMACS profile, concomitant right ventricular assist device, absence of any concomitant procedures, and pre-LVAD dialysis. Features that were commonly important in the 1-year ML mortality model included age, creatinine, albumin, weight, height, prior cardiac operation, and pre-implant dialysis.

**Table 2:** Calibration metrics of the logistic regression and machine learning models for 90-day and 1-year mortality in the testing cohorts.

**90-Day Mortality**	***Logistic Regression***	***Machine Learning***
Observed-to-Expected Ratio	0.950	0.966
Calibration-in-the-Large	-0.067	-0.043
Slope of Calibration Curve	0.846	1.288
**1-Year Mortality**	***Logistic Regression***	***Machine Learning***
Observed-to-Expected Ratio	1.065	1.074
Calibration-in-the-Large	0.091	0.101
Slope of Calibration Curve	0.930	1.248

**Table 3:** Area under receiver-operating-characteristic curve of the logistic regression and machine learning models for 90-day and 1-year mortality in the testing cohorts.

**90-Day Mortality**	***Logistic Regression***	***Machine Learning***	***P-value***
AUROC	0.707 (0.683–0.730)	0.740 (0.717–0.762)	<0.001
**1-Year Mortality**	***Logistic Regression***	***Machine Learning***	
AUROC	0.691 (0.673–0.710)	0.714 (0.695–0.734)	<0.001

### Concordance analysis

For 90-day mortality, most patients (66.6%; n = 2,683) in the testing cohort had concordant estimated risk between LR and ML. Calibration metrics for the LR 90-day mortality model were improved when ML provided concordant prediction, particularly with regards to slope of the calibration curve (**Table 4**). There were minimal differences in calibration metrics for the ML 90-day mortality model in cases of discordance versus concordance with LR. Similar trends were observed with the 1-year mortality models (**Table 4**).

**Table 4:** Comparison of calibration metrics in the testing cohorts in cases of concordant versus discordant risk prediction between logistic regression and machine learning approaches.

LOGISTIC REGRESSION		
**90-Day Mortality**	***Discordant (33*.*4%; n = 1*,*347)***	***Concordant(66*.*6%; n = 2*,*683)***
Observed-to-Expected Ratio	0.918	0.962
Calibration-in-the-Large	-0.099	-0.053
Slope of Calibration Curve	0.165	0.925
**1-Year Mortality**	***Discordant (28*.*2%; n = 1*,*137)***	***Concordant (71*.*8%; n = 2*,*893)***
Observed-to-Expected Ratio	1.042	1.072
Calibration-in-the-Large	0.054	0.106
Slope of Calibration Curve	0.386	0.990
**MACHINE LEARNING**		
**90-Day Mortality**	***Discordant (33*.*4%; n = 1*,*347)***	***Concordant (66*.*6%; n = 2*,*683)***
Observed-to-Expected Ratio	0.844	1.017
Calibration-in-the-Large	-0.194	0.021
Slope of Calibration Curve	0.990	1.304
**1-Year Mortality**	***Discordant (28*.*2%; n = 1*,*137)***	***Concordant (71*.*8%; n = 2*,*893)***
Observed-to-Expected Ratio	1.014	1.094
Calibration-in-the-Large	0.019	0.132
Slope of Calibration Curve	1.060	1.262

Substantial differences in AUROC were demonstrated in cases of concordance versus discordance across all models (**Table 5**). Moreover, 90-day mortality AUROC increased from 0.536 to 0.752 for the LR model in cases of discordance versus concordance with ML, respectively (p<0.001). The LR 1-year mortality AUROC similarly increased from 0.555 to 0.726 with concordance with ML (p<0.001). The ML 90-day mortality AUROC improved from 0.623 to 0.772 when concordant with LR predicted risk (p<0.001). Similarly, 1-year ML mortality AUROC improved from 0.613 to 0.742 in cases of concordance (p<0.001). When comparing the AUROC of the concordant cases to overall AUROC for the entire cohort, the improvements in performance were again noted to be significant across the comparisons (each p<0.001 for both LR and ML for both 90-day and 1-year mortality).

**Table 5:** Comparison of area under receiver-operating-characteristic curves in the testing cohorts in cases of concordant versus discordant risk prediction between logistic regression and machine learning.

LOGISTIC REGRESSION			
**90-Day Mortality**	***Discordant (33*.*4%; n = 1*,*347)***	***Concordant (66*.*6%; n = 2*,*683)***	***P-value***
AUROC	0.536 (0.485–0.587)	0.752 (0.727–0.778)	<0.001
**1-Year Mortality**	***Discordant (28*.*2%; n = 1*,*137)***	***Concordant (71*.*8%; n = 2*,*893)***	
AUROC	0.555 (0.514–0.596)	0.726 (0.705–0.747)	<0.001
**MACHINE LEARNING**			
**90-Day Mortality**	***Discordant (33*.*4%; n = 1*,*347)***	***Concordant (66*.*6%; n = 2*,*683)***	***P-value***
AUROC	0.623 (0.576–0.670)	0.772 (0.748–0.797)	<0.001
**1-Year Mortality**	***Discordant (28*.*2%; n = 1*,*137)***	***Concordant (71*.*8%; n = 2*,*893)***	
AUROC	0.613 (0.574–0.652)	0.742 (0.722–0.762)	<0.001

The observed rates of 90-day mortality in the validation cohort fell within the range of predicted risk in cases of concordance between LR and ML, from 3.4% in low risk to 9.7% in intermediate risk to 27.5% 90-day mortality in high risk (**Table 6**). In the other 6 permutations of discordant risk prediction, the observed rates of 90-day mortality were within the range of predicted risk by ML but not within the range of predicted risk by the LR model (**Table 6**).

**Table 6:** Observed rates of 90-day mortality in the validation set based on permutations of predicted risk by the LR and ML models, with gray shaded boxes representing areas of concordance.

	LR Model →	Low (<7.6%)	Intermediate (7.6–14.0%)	High (>14.0%)
**ML Model ↓**				
**Low (<7.6%)**		**3.4% (n = 844)**	4.2% (n = 189)	0% (n = 10)
**Intermediate (7.6–14.0%)**		8.1% (n = 459)	**9.7% (n = 832)**	10.2% (n = 325)
**High (>14.0%)**		14.3% (n = 42)	15.4% (n = 324)	**27.5% (n = 1,007)**

Similarly, observed rates of 1-year mortality in the validation set fell within the range of predicted risk in cases of concordance between LR and ML, ranging from 9.5% in low risk to 20.9% in intermediate risk to 43.8% in high risk cases (**Table 7**). Unlike 90-day mortality, there was a mix of discordant permutations where observed rates of 1-year mortality fell within range of the LR prediction and others that fell within the range of ML prediction (**Table 7**).

**Table 7:** Observed rates of 1-year mortality in the validation set based on permutations of predicted risk by the LR and ML models, with gray shaded boxes representing areas of concordance.

	LR Model →	Low (<15.6%)	Intermediate (15.6–25.1%)	High (>25.1%)
**ML Model ↓**				
**Low (<15.6%)**		**9.5% (n = 961)**	12.7% (n = 221)	5.6% (n = 18)
**Intermediate (15.6–25.1%)**		18.5% (n = 368)	**20.9% (n = 870)**	27.4% (n = 263)
**High (>25.1%)**		13.3% (n = 15)	26.7% (n = 251)	**43.8% (n = 1,062)**

## Discussion

This study demonstrates the potential utility of employing ML approaches to risk prediction in LVAD therapy. While both LR and ML models were well-calibrated, ML was associated with a statistically significant improvement in discriminatory performance for both 90-day and 1-year mortality. These trends are similar to what has been observed in analyses of general adult cardiac surgical procedures where ML has been associated with improved predictive capability in risk modeling [5, 9, 10]. The implications of the current study are important as risk modeling plays a critical role in determining LVAD surgical candidacy, selection of type of advanced heart failure therapy, patient counseling and prognostication, and quality improvement.

Risk models using larger datasets such as INTERMACS can have limited predictive performance for multiple reasons. Foremost, as with most national data repositories, the variables that are collected are ideally easily extractable and routinely collected in the clinical course of the patient. If these criteria are not met, those variables tend to be excluded from registry data collection or can have high degrees of missing data which limit the ability to use that variable in risk modeling. There are likely predictive trends and data related to both the patient and their disease course that are informative of outcomes including mortality, however, for these reasons are not captured.

Another factor that limits predictive capability is the outcome itself. All-cause mortality by definition can stem from a variety of clinical pathways and etiologies, each with different sets of predictive risk factors. An increasing body of work has demonstrated that distinct sequences of adverse events occur during LVAD support, many of which have distinct predictors of occurrence and varying likelihoods of subsequent death [11, 12]. Interestingly, the AUROC of both the LR and ML models was greater for 90-day mortality than it was for 1-year mortality. This suggests that pre-implant risk factors are likely less predictive of survival as time progresses from the date of LVAD surgery. Other longitudinal factors such as compliance, development and severity of adverse events such as gastrointestinal bleeding and infection, device malfunction or thrombosis, declining right heart function, and blood pressure management are likely to have increasing weight in defining the clinical course of LVAD patients with progression of time on support.

### Prior work

Prior work has evaluated the performance of various risk modeling strategies in predicting survival after LVAD implantation. Cowger and colleagues evaluated multiple risk indices including the HeartMate II risk score, the model for end-stage liver disease, and the destination therapy risk score in a cohort of durable LVAD recipients [4]. Although the risk scores had reasonable AUROC for 90-day mortality in the derivation cohorts (0.60–0.77), the AUROC dropped substantially in the validation sets (0.59–0.64). A study utilizing Bayesian approaches to predict survival after LVAD surgery in the INTERMACS dataset displayed AUROCs of 0.71 and 0.70 for 90-day and 1-year mortality, respectively [13]. Our ML model compares favorably with this prior work, particularly for 90-day mortality prediction where the AUROC was 0.74.

### Concordance

In the current study we also demonstrate that evaluating concordance in predicted risk between various modeling approaches can yield important insights. Moreover, there were profound differences in AUROC in subsets where LR and ML had discordant risk prediction versus concordant risk prediction. Furthermore, the observed rates of mortality in the validation cohorts were within the range of predicted risk when there was agreement between LR and ML in estimated tertiles of risk. This was similar to what was demonstrated in a study utilizing both LR and XGBoost to predict acute kidney injury after percutaneous coronary intervention [14]. Further exploration into mechanisms of differing risk prediction between LR and ML in discordant patients may yield insights into why these models perform much more poorly in discordant patients.

These findings may have important implications in how risk models are incorporated in LVAD therapy. For example, surgical risk is typically conveyed as an absolute percent risk to patients. However, our concordance analysis suggests that the confidence in our estimates of risk are substantially different in concordant versus discordant patients. Therefore, it may be prudent to convey a range of risk or our confidence in estimated risk to patients, rather than a simple percentage. Similarly, for programmatic evaluation, one could argue that if such risk models are utilized to derive expected outcomes for calculating observed-to-expected ratios, that patients with discordant risk prediction between LR and ML could be excluded or weighted less heavily in such calculations.

### Study limitations

This is a retrospective study and therefore has inherent limitations related to the study design. The INTERMACS database is a multicenter registry and is subject to errors in data entry. There may be additional variables that can improve the predictive performance of risk models for LVADs that are not included in INTERMACS and therefore could be not evaluated. This includes temporal changes that may have occurred during the study period, including changes in patient management, surgical technique, and specific device types. There are a cadre of other ML algorithms besides XGBoost that were not assessed in this analysis but may have differing impacts on mortality prediction. Comparison to existing risk models such as the HeartMate II risk score were not possible due to some components of these pre-existing models being missing from the version of the INTERMACS dataset used for this study.

## Conclusions

This analysis utilized the INTERMACS database to develop and compare risk models for 90-day and 1-year mortality following primary durable LVAD implantation using LR and ML approaches. The ML models derived using the XGBoost algorithm were well-calibrated and had improved discriminatory capability as compared to LR. In addition, there were profound differences in model performance in patients with concordant versus discordant LR and ML estimated risk. These findings suggest that ML may have an important role in risk prediction in LVAD therapy, both independently as well as an adjunct to traditional modeling approaches such as LR.
